# Dedifferentiated Gastric Gastrointestinal Stromal Tumor with Rapid Growth Mimicking Other Malignancies: A Case Report

**DOI:** 10.70352/scrj.cr.26-0416

**Published:** 2026-09-05

**Authors:** Ken Iijima, Tsuyoshi Takahashi, Yukinori Kurokawa, Takuro Saito, Takaomi Hagi, Shigeto Nakai, Kota Momose, Kotaro Yamashita, Koji Tanaka, Tomoki Makino, Kiyokazu Nakajima, Yoshiharu Genko, Masako Kurashige, Eiichi Morii, Hidetoshi Eguchi, Yuichiro Doki

**Affiliations:** 1 Department of Gastroenterological Surgery, Osaka University Graduate School of Medicine, Suita, Osaka, Japan; 2 Department of Pathology, Osaka University Graduate School of Medicine, Suita, Osaka, Japan

**Keywords:** gastrointestinal stromal tumor (GIST), dedifferentiation, gastric GIST, tyrosine kinase inhibitor–naïve, KIT-negative, DOG1-negative

## Abstract

**INTRODUCTION:**

Gastrointestinal stromal tumors (GISTs) are diagnosed based on morphological and immunohistochemical findings. However, in rare instances, GISTs may undergo morphological and immunophenotypic dedifferentiation, making diagnosis challenging. Here, we report a rare case of a rapidly growing gastric GIST with a dedifferentiated component that posed a diagnostic challenge, together with a review of the literature.

**CASE PRESENTATION:**

A woman in her 70s presented with abdominal distention and was found to have a rapidly enlarging 21 cm gastric tumor. Endoscopic US-guided biopsy showed Class V cytology; however, a definitive histologic diagnosis could not be established, and immunohistochemical staining was negative for all examined markers. Distal gastrectomy and partial resection of the transverse colon were performed. Histologically, the tumor consisted of a conventional GIST component with immunohistochemically positive atypical spindle cells and a dedifferentiated component with immunohistochemically negative pleomorphic atypical cells. The tumor was diagnosed as dedifferentiated GIST. Anastomotic recurrence and peritoneal dissemination developed 11 months postoperatively.

**CONCLUSIONS:**

Here, we report an extremely rare case with findings most compatible with dedifferentiated gastric GIST occurring without prior TKI therapy. GIST should not be excluded even when both KIT and DOG1 are negative; a careful integrated diagnosis based on histopathological findings, imaging findings, and the clinical course is essential.

## Abbreviations

CD34
: cluster of differentiation 34
CK AE1/AE3
: cytokeratin AE1/AE3
DOG1
: discovered on GIST-1
EMA
: epithelial membrane antigen
GATA3
: GATA-binding protein 3
GIST
: gastrointestinal stromal tumor
hCG
: human chorionic gonadotropin
HMB45
: human melanoma black 45
KIT
: tyrosine-protein kinase KIT (CD117)
LCA
: leukocyte common antigen
MDM2
: mouse double minute 2
MyoD1
: myogenic differentiation 1 protein
PAX8
: paired box gene 8
PDGFRA
: platelet-derived growth factor receptor alpha
S100
: S100 protein
SOX10
: SRY-box transcription factor 10
STAT6
: signal transducer and activator of transcription 6
TKI
: tyrosine kinase inhibitor
αSMA
: alpha-smooth muscle actin

## INTRODUCTION

GISTs are the most common mesenchymal neoplasms of the gastrointestinal tract and are believed to arise from interstitial cells of Cajal or their precursor cells. Histologically, GISTs typically show a spindle or epithelioid cell morphology in hematoxylin and eosin-stained sections. Immunohistochemically, most GISTs express KIT (CD117).^1)^ DOG1 is also a highly sensitive and specific marker for GISTs and may be present in KIT-negative tumors.^1,2)^ In routine practice, GISTs are diagnosed based on KIT and DOG1 expression.

A subset of GISTs shows dedifferentiation, characterized by morphological and immunophenotypic features distinct from those of conventional GISTs. Most reported cases occur as secondary changes after treatment with TKIs. In dedifferentiated GISTs, loss of KIT or DOG1 expression and morphological alterations may occur, leading to potential diagnostic pitfalls.^3,4)^

Although dedifferentiated GISTs without prior TKI therapy have been reported, they are exceedingly rare.^3)^ Herein, we report a rare case of gastric dedifferentiated GIST without prior TKI therapy and review the relevant literature.

## CASE PRESENTATION

A woman in her 70s presented with abdominal distention. At the referral hospital, a palpable mass was found in the upper abdomen. CT revealed an intra-abdominal mass, and the patient was referred to our department for further evaluation and treatment.

Her medical history included hypertension and glaucoma. The Eastern Cooperative Oncology Group performance status was 1. She was 148 cm tall and weighed 50 kg (BMI, 22.8); however, she had lost 7 kg during the preceding 2 months. Her body temperature was 38.0°C at presentation.

Laboratory examination showed an elevated inflammatory response, with a white blood cell count of 16980/µL and a C-reactive protein level of 16.7 mg/dL. Liver enzyme levels were also elevated (aspartate aminotransferase, 115 U/L; alanine aminotransferase, 158 U/L), and hypoalbuminemia was present (albumin, 1.5 g/dL). Tumor marker levels were within normal limits.

**Fig. 1:**
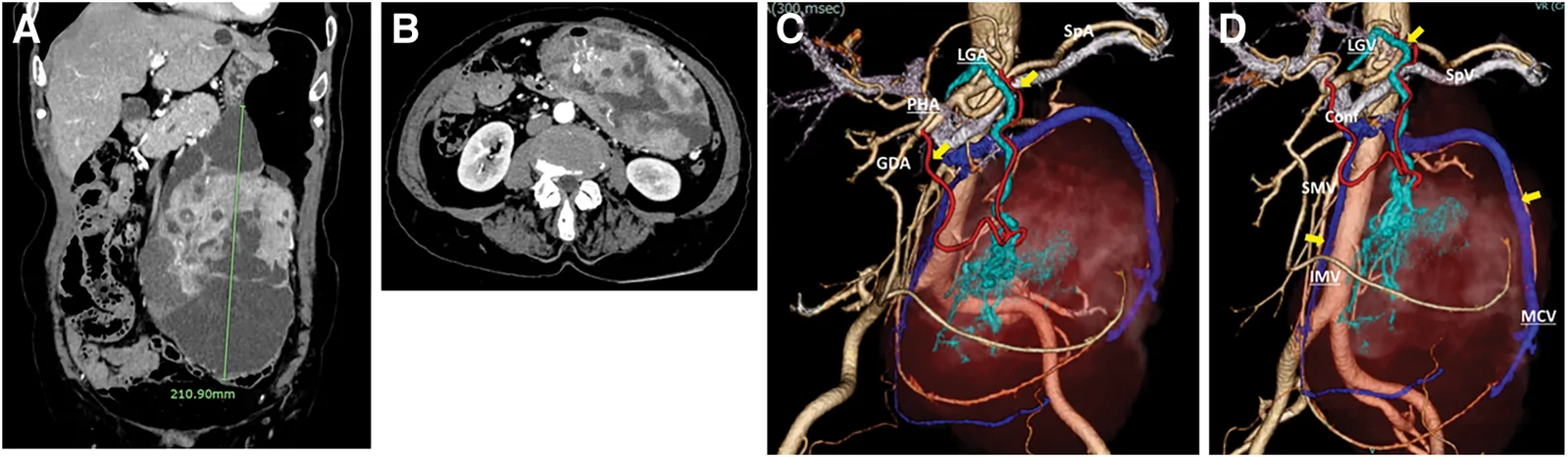
Contrast-enhanced CT findings of the tumor. (**A**) Contrast-enhanced CT shows a large intra-abdominal mass measuring 21 cm in diameter. (**B**) A small aneurysmal structure is identified within the tumor. (**C**) Arterial-phase image demonstrating tumor supply from the left gastric and proper hepatic arteries, indicated by yellow arrows. (**D**) Venous-phase image showing tumor drainage into the left gastric, middle colic, and inferior mesenteric veins, indicated by yellow arrows. Conf, confluence of the superior mesenteric vein and splenic vein; GDA, gastroduodenal artery; IMV, inferior mesenteric vein; LGA, left gastric artery; LGV, left gastric vein; MCV, middle colic vein; PHA, proper hepatic artery; SMV, superior mesenteric vein; SpA, splenic artery; SpV, splenic vein

**Fig. 2:**
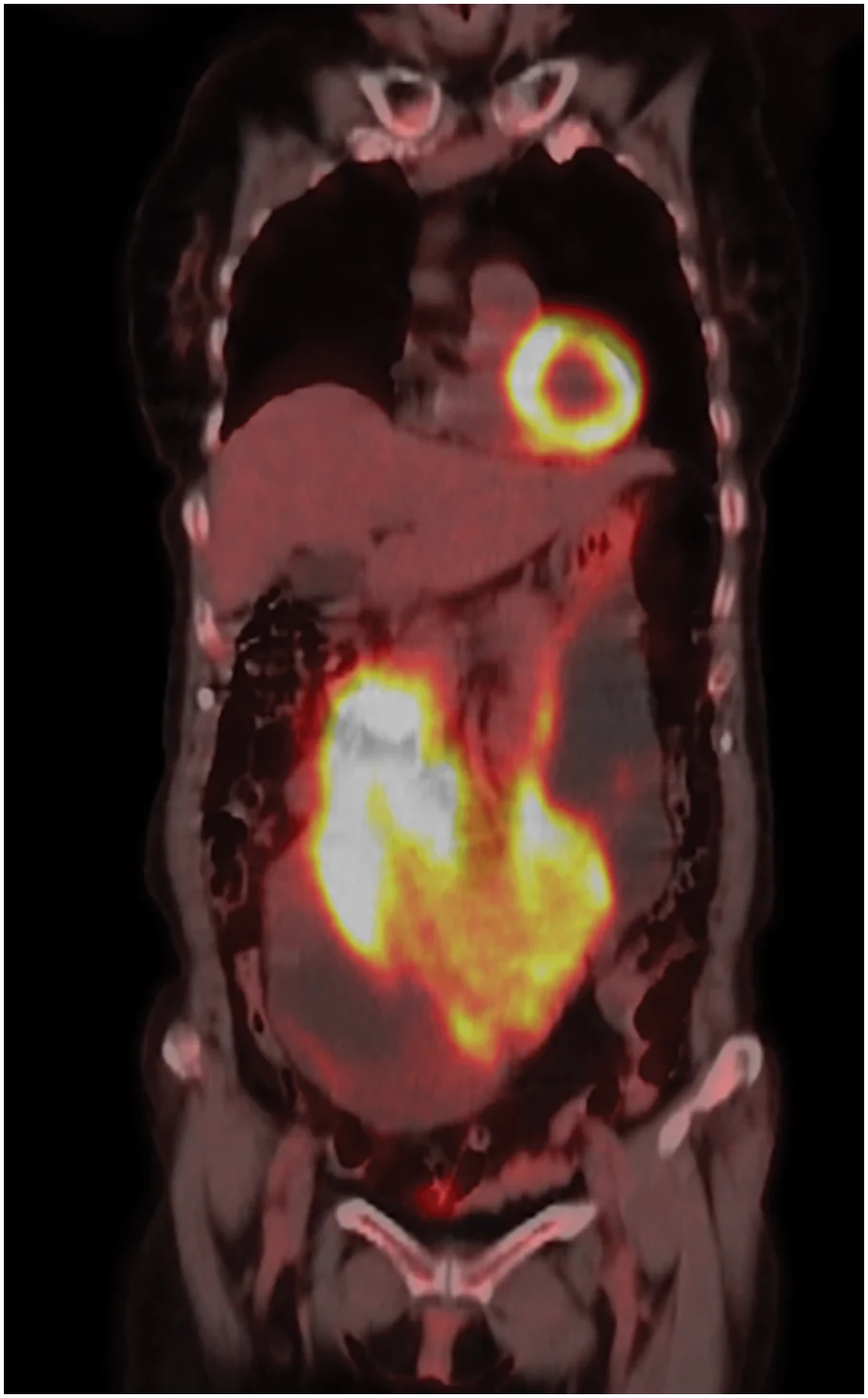
PET-CT findings of the tumor. PET–CT shows increased fluorodeoxyglucose uptake within the intra-abdominal tumor, without lymph node or distant metastases.

Contrast-enhanced CT demonstrated a 21 cm intra-abdominal mass (**Fig. 1A**) with a small aneurysm identified within the tumor (**Fig. 1B**). The feeding arteries were the left gastric and proper hepatic arteries (**Fig. 1C**), and the drainage veins included the left gastric, middle colic, and inferior mesenteric veins (**Fig. 1D**). PET-CT demonstrated abnormal fluorodeoxyglucose uptake within the tumor (**Fig. 2**) without evidence of lymph node or distant metastases.

**Fig. 3:**
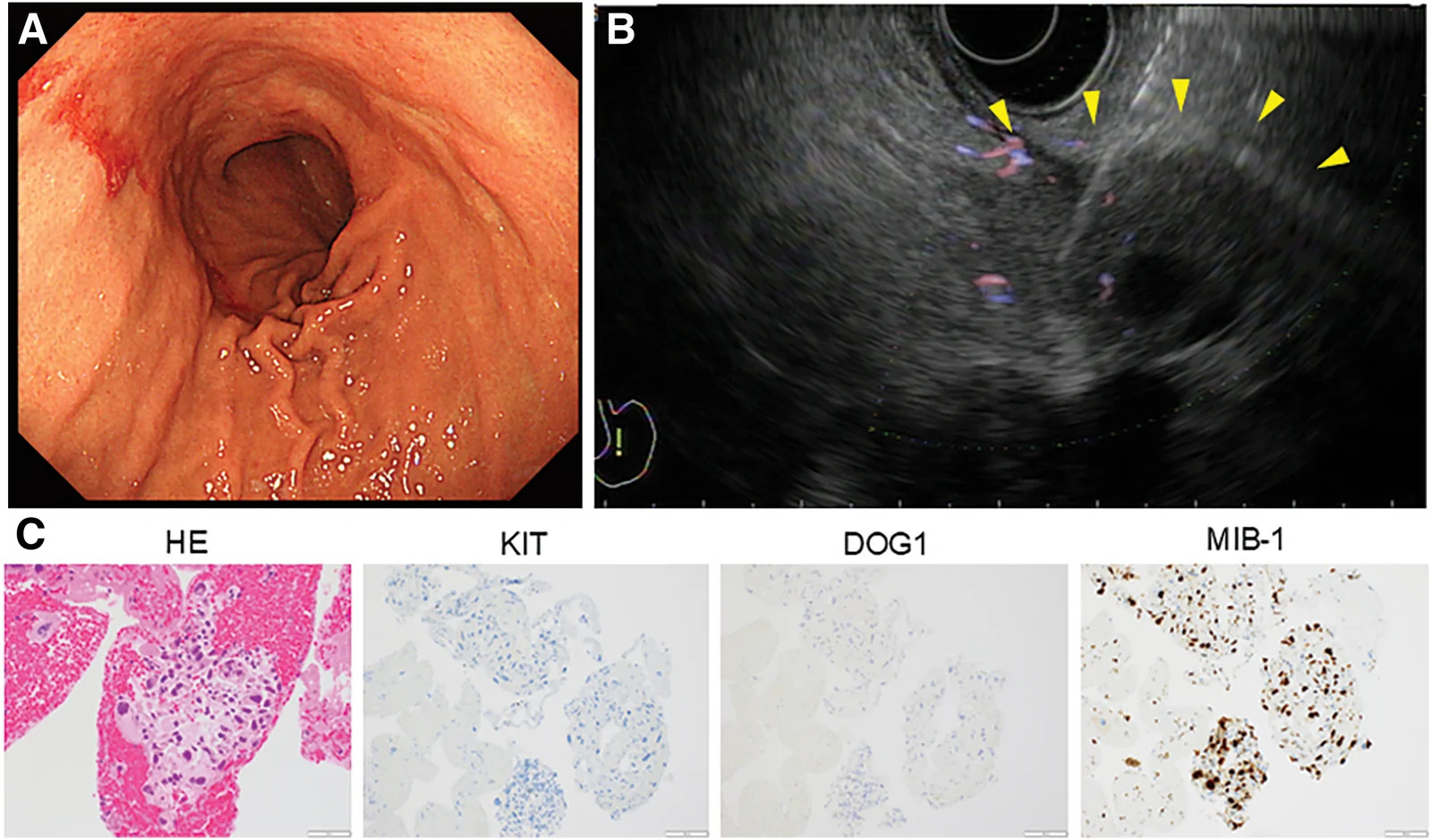
Endoscopic and endoscopic ultrasonography findings and fine-needle aspiration. (**A**) Upper gastrointestinal endoscopic ultrasonography shows no mucosal involvement of the tumor. (**B**) Endoscopic ultrasonography shows a mass with both solid and fluid components (yellow arrowheads). (**C**) Cytological and immunohistochemical findings from preoperative biopsy specimens. Fine-needle aspiration cytology with hematoxylin and eosin staining shows atypical malignant cells (scale bar = 50 μm). Immunohistochemical staining is negative for KIT and DOG1, with a Ki-67 labeling index of 80% (scale bar = 100 μm). DOG1, discovered on GIST-1; HE, hematoxylin-eosin staining; KIT, tyrosine-protein kinase KIT (CD117)

Upper gastrointestinal endoscopic ultrasonography revealed no mucosal involvement of the tumor (**Fig. 3A**) and a mass containing both solid and fluid components (**Fig. 3B**). The overall configuration of the lesion and its origin could not be fully assessed. Part of the tumor was hyperechoic and showed a to-and-fro pattern, suggesting an intratumoral hemorrhage. Fine-needle aspiration of the solid component yielded cytological findings consistent with malignancy; however, a definitive pathological diagnosis could not be established (**Fig. 3C**). Immunohistochemical staining was negative for all examined markers, including KIT, DOG1, and CD34 (CK AE1/AE3, S100, αSMA, desmin, LCA, HMB45, MelanA, synaptophysin, claudin-4, GATA3, PAX8, inhibin-α, CAM5.2, calretinin, D2-40, caldesmon, MDM2, myogenin, MyoD1, and hCG). The Ki-67 labeling index was 80%.

Preoperatively, an unclassifiable high-grade malignancy, such as an undifferentiated sarcoma, was suspected because the biopsy specimen was negative for KIT, DOG1, and CD34 and demonstrated a high Ki-67 labeling index. The patient also had severe preoperative malnutrition, as evidenced by a 7-kg weight loss over 2 months and marked hypoalbuminemia, which was considered to be primarily attributable to the rapidly enlarging tumor and associated systemic inflammation. Given the aggressive tumor growth, progressive malnutrition, and the risk of further clinical deterioration, prolonged preoperative nutritional optimization was not considered feasible, and surgical resection was performed for both diagnostic and therapeutic purposes.

During laparotomy, the tumor was found to arise from the posterior wall of the gastric body; therefore, it was considered to be of gastric origin. A distal gastrectomy was performed. Because of partial invasion of the transverse colon, partial transverse colectomy was also performed. The patient had severe preoperative malnutrition, including marked hypoalbuminemia, and bowel edema was observed intraoperatively. Given the substantial risk of anastomotic leakage associated with these conditions, primary intestinal anastomosis was avoided, and a colostomy and enterostomy were created. The operative time was 3 h 56 min, and blood loss was 830 mL.

**Fig. 4:**
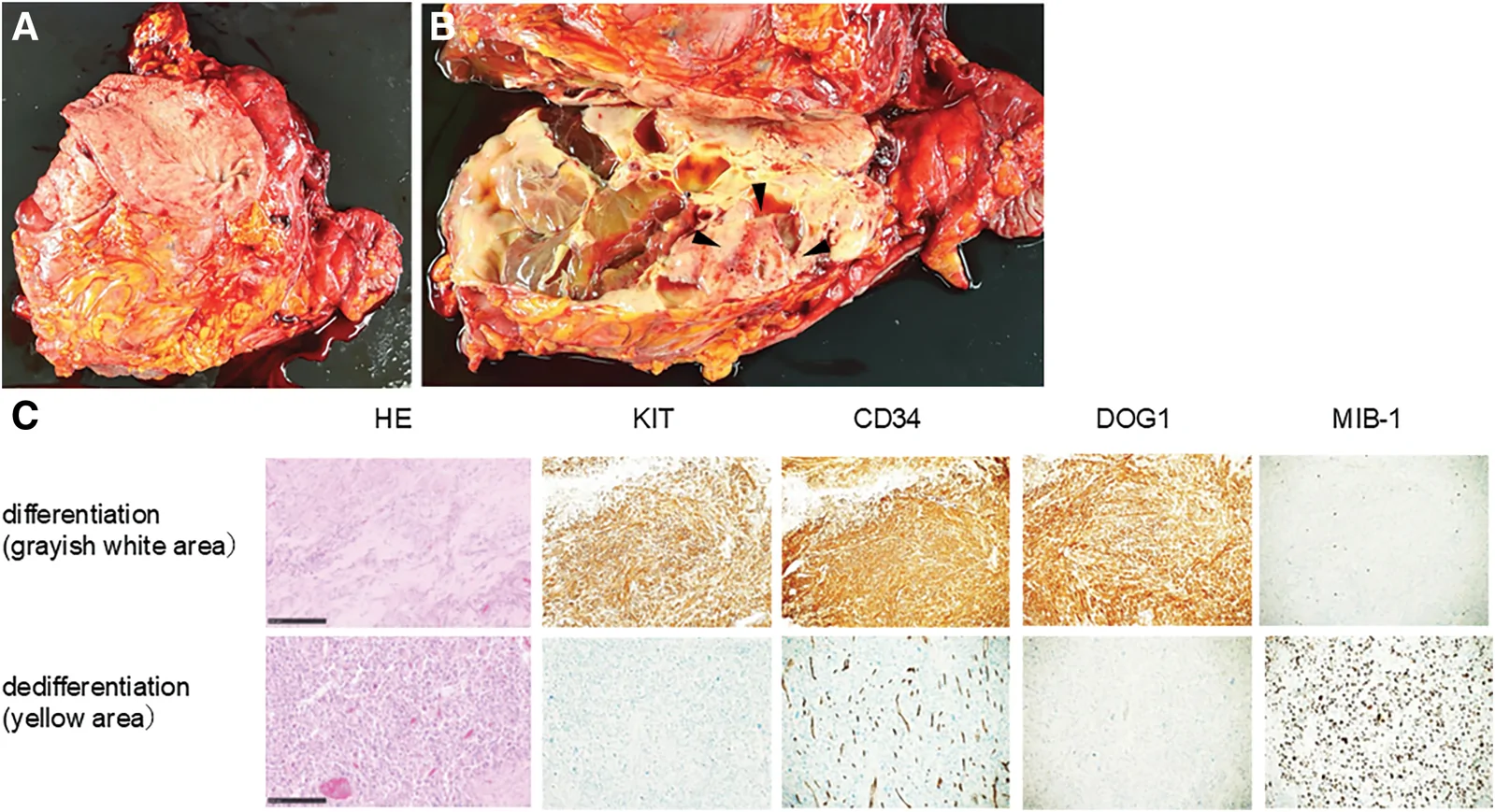
Gross and histologic findings of the resected specimen. (**A**) Gross photograph of the resected specimen shows a tumor extending from the serosal surface of the stomach to that of the transverse colon. (**B**) The cut surface shows a predominantly yellowish area with hemorrhage and a focal gray-white area (black arrowheads). (**C**) Histological findings of the differentiated and dedifferentiated components (scale bar = 100 μm). The gray-white area shows spindle cells positive for KIT, CD34, and DOG1, with a low Ki-67 labeling index of 2%. In contrast, the yellowish area shows highly atypical multinucleated cells that are negative for immunohistochemical markers, with a Ki-67 labeling index of 50%. CD34, cluster of differentiation 34; DOG1, discovered on GIST-1; HE, hematoxylin-eosin staining; KIT, tyrosine-protein kinase KIT (CD117)

Gross examination of the resected specimen revealed a tumor extending from the serosal surface of the stomach to that of the transverse colon (**Fig. 4A**). Macroscopically, most of the tumor was yellowish with hemorrhage, and a focal gray-white area was identified (**Fig. 4B**). Histologically, the yellowish area, which comprised most of the tumor, consisted of highly atypical cells with enlarged pleomorphic nuclei, abundant eosinophilic cytoplasm, frequent multinucleation, and bizarre nuclei. In contrast, the gray-white area showed proliferation of atypical spindle cells with oval nuclei arranged in an interlacing pattern within a hyalinized collagenous stroma. The spindle cells in the gray-white area were positive for KIT, CD34, and DOG1, consistent with GIST. Immunohistochemical staining for other markers (CK AE1/AE3, EMA, S100, αSMA, desmin, SOX10, STAT6, and MDM2) was negative. The Ki-67 labeling index was 2%. Conversely, atypical multinucleated cells in the yellowish area were negative for all examined immunohistochemical markers, and the Ki-67 labeling index was 50% (**Fig. 4C**), consistent with a dedifferentiated component. Genetic analyses revealed no pathogenic *KIT or PDGFRA* mutations in either the differentiated or dedifferentiated components.

Adjuvant therapy was not administered postoperatively because clear evidence supporting adjuvant TKI therapy in wild-type or dedifferentiated GIST is lacking. Eleven months after the initial resection, contrast-enhanced CT revealed peritoneal dissemination, indicating disease recurrence. Although the absence of pathogenic KIT or PDGFRA mutations suggested a lower likelihood of response, imatinib was initiated in accordance with the Japanese clinical practice guidelines for GIST. Because limited therapeutic efficacy was anticipated, treatment response was assessed early. CT performed 1 month after treatment initiation demonstrated progressive disease. Surgical resection of the recurrent lesions was performed. At the time of this report, the patient is alive without recurrence.

## DISCUSSION

GISTs typically show a spindle or epithelioid cell morphology on hematoxylin and eosin-stained sections, and immunohistochemical staining is usually positive for KIT and DOG1. Morphological and immunohistochemical findings form the basis of diagnosis.^3,5)^ Dedifferentiation refers to the loss of specific gene expression and regression from a specialized tissue phenotype to a more primitive developmental state.^4)^ In 2005, Pauwels et al. first reported dedifferentiated GIST after imatinib administration. They defined dedifferentiated GIST as a transformation from conventional KIT-positive GIST to a high-grade KIT-negative tumor, with loss of the histologic features of the primary tumor.^6)^ In 2013, Antonescu et al. reported the presence of dedifferentiated GIST in patients without a history of TKI treatment. Regardless of prior TKI therapy, dedifferentiated GISTs should be considered in the differential diagnosis, even when immunohistochemical staining is negative.^3)^ The discrepancy in the Ki-67 labeling index between the biopsy and resected specimens may reflect intratumoral heterogeneity and sampling bias, with the biopsy specimen potentially sampling a particularly proliferative area of the tumor.

Reports of dedifferentiated gastric GISTs without prior imatinib therapy, as in this case, are rare. A PubMed search using the keywords “gastrointestinal stromal tumor” and “dedifferentiation” identified 8 reported cases of dedifferentiated gastric GISTs, including the present case, in which conventional KIT-positive components coexisted with dedifferentiated KIT-negative components in patients without prior imatinib treatment (**Table 1**).^3,7–9)^ The mean patient age was 52 years, the male-to-female ratio was 5:3, and the mean tumor size was 14.4 cm. Histopathological examination revealed an undifferentiated appearance in the dedifferentiated component in 7 cases, and numerous mitotic figures were observed in all cases. Although DOG1 was not assessed in all cases, the dedifferentiated components were negative for KIT, CD34, and DOG1. Dedifferentiated gastric GISTs are considered highly malignant, as synchronous or metachronous metastases were observed in 7 of 8 cases, suggesting an aggressive clinical course. In the present case, the recurrent tumor showed dedifferentiated components, which likely contributed to disease progression and unfavorable outcomes.

**Table 1:** Reported cases of gastric GIST with dedifferentiated components in patients without prior molecular targeted therapy

Case	Age/Sex	Primary site	Size	Pathological morphology (differentiation/dedifferentiation)	Mitotic count^*^(differentiation/dedifferentiation)	Immunohistochemical findings^#^ (differentiation/dedifferentiation)			Metastasis
						KIT	CD34	DOG1	
1^3)^	40/F	Stomach	8	Spindle/Dedifferentiated (Anaplastic)	1/>10	P/N	N/N	Unknown	Peritoneal
2^3)^	55/M	Stomach	18	Spindle/Dedifferentiated (Anaplastic)	>10/>10	P/N	P/N	Unknown	No
3^3)^	48/M	Stomach	5.5	Mixed/Dedifferentiated (Anaplastic)	1/>10	P/N	P/N	Unknown	Liver, Peritoneal
4^3)^	53/M	Stomach	7	Spindle/Dedifferentiated (Anaplastic)	>10/>10	P/N	P/N	Unknown	Peritoneal
5^7)^	51/M	Stomach	11	Spindle/Dedifferentiated (Fibroblastic)	>10/>10	P/N	P/N	P/N	Liver
6^8)^	61/M	Stomach	30	Spindle/Dedifferentiated (Anaplastic)	– >35	P/N	P/N	P/N	Intra- and extra-abdominal
7^9)^	32/F	Stomach	10	Epithelioid/Dedifferentiated (Anaplastic)	4/75	P/N	P/N	P/N	Lymph nodes
Our case	73/F	Stomach	26	Spindle/Dedifferentiated (Anaplastic)	0/>10	P/N	P/N	P/N	Peritoneal

^*^Mitotic count was evaluated as the number of mitoses per 50 HPFs.

^#^P indicates positive, and N indicates negative immunohistochemical staining.

CD34, cluster of differentiation 34; DOG1, discovered on GIST-1; F, female; GIST, gastrointestinal stromal tumor; HPFs, high-power fields; KIT, tyrosine-protein kinase KIT (CD117); M, male

From a molecular genetic perspective, previous studies have shown that the differentiated and dedifferentiated components of dedifferentiated GISTs generally share the same driver alterations, suggesting a common clonal origin and morphological transformation during progression.^3)^ Additionally, some studies have described *KIT* copy number abnormalities in the dedifferentiated component, suggesting that dedifferentiation may be associated with genetic instability.^3)^ In the present case, mutational analysis of *KIT* exons 8, 9, 11, 13, 14, 17, and 18 was performed for both the differentiated and dedifferentiated components, and no pathogenic mutations were identified in either component. While the identical wild-type status in both components supports a shared origin, this finding alone is insufficient to conclusively demonstrate clonality. The conventional GIST component and the undifferentiated component were closely associated within the same tumor, and loss of GIST-associated immunophenotypic markers in the undifferentiated component has been reported in previously described dedifferentiated GISTs. Therefore, the findings were considered most compatible with dedifferentiated GIST. Furthermore, the 2 components did not form clearly separate expansile lesions. Nevertheless, because molecular clonality between the 2 components was not demonstrated, a collision tumor composed of conventional GIST and an undifferentiated pleomorphic sarcoma-like tumor cannot be completely excluded.^10,11)^

Dedifferentiated GISTs are increasingly recognized as a phenotype associated with resistance to imatinib therapy.^3)^ Previous studies have reported that dedifferentiation often emerges during or after treatment with imatinib and is accompanied by marked morphological changes as well as loss or reduction of KIT and DOG1 expression, suggesting a shift away from KIT-dependent oncogenic signaling.^12)^ Although imatinib resistance in GIST is generally attributed to secondary genetic mutations, the molecular background of dedifferentiated GIST appears heterogeneous,^13)^ and the mechanisms underlying dedifferentiation and therapeutic resistance remain incompletely understood.^4)^

Although several TKIs, including sunitinib, regorafenib, and ripretinib, have been approved for advanced or recurrent GIST,^14)^ their efficacy in dedifferentiated GIST remains unclear.^4)^ In the present case, adjuvant therapy was not administered because the tumor was classified as a wild-type GIST. Although the absence of pathogenic KIT or PDGFRA mutations suggested a lower likelihood of response, imatinib was initiated after recurrence in accordance with the Japanese clinical practice guidelines for GIST. However, early assessment demonstrated progressive disease, necessitating prompt surgical resection of the recurrent lesion. This clinical course suggests that dedifferentiated components may exhibit limited sensitivity to imatinib. Dedifferentiated GISTs often exhibit a rapid disease course and are frequently associated with widespread metastases,^15)^ rendering surgical resection challenging in many cases. In this context, the clinical course observed in the present case, in which surgical resection was feasible after recurrence, should be interpreted with caution and may not be generalizable. Given the absence of standardized therapeutic strategies, further elucidation of the molecular mechanisms underlying dedifferentiation is essential to develop effective treatments for this rare condition.

This case has several limitations. First, molecular clonality between the conventional GIST and undifferentiated components was not demonstrated. Therefore, although the close spatial relationship of the 2 components within the same tumor and the loss of GIST-associated immunophenotypic markers in the undifferentiated component supported the diagnosis of dedifferentiated GIST, a collision tumor composed of conventional GIST and an undifferentiated pleomorphic sarcoma-like tumor could not be completely excluded. Second, immunohistochemical staining for SDHB and sequencing analyses of other genes associated with KIT/PDGFRA wild-type GIST, including BRAF and NF1, were not performed. Therefore, SDH-deficient GIST and BRAF- or NF1-associated GIST could not be completely excluded.

## CONCLUSIONS

In conclusion, this was an extremely rare case with findings most compatible with dedifferentiated gastric GIST occurring in the absence of prior TKI therapy. GIST should not be excluded solely because both KIT and DOG1 are negative; a comprehensive assessment integrating histopathological findings, imaging findings, and the clinical course is essential, with dedifferentiated GIST included in the differential diagnosis. Because dedifferentiated GISTs have rarely been reported, their pathobiology and responsiveness to treatment remain poorly understood. Further accumulation of cases is required to better characterize their biological features and establish appropriate diagnostic criteria.

## DECLARATIONS

### Availability of data and materials

The data supporting the findings of this study are available from Tsuyoshi Takahashi upon request. The data are not publicly available because they contain information that could compromise participant privacy.
